# BRAF inhibition curtails IFN‐gamma‐inducible PD‐L1 expression and upregulates the immunoregulatory protein galectin‐1 in melanoma cells

**DOI:** 10.1002/1878-0261.12695

**Published:** 2020-05-19

**Authors:** Patryk Górniak, Maja Wasylecka‐Juszczyńska, Iwona Ługowska, Piotr Rutkowski, Anna Polak, Maciej Szydłowski, Przemysław Juszczyński

**Affiliations:** ^1^ Department of Experimental Hematology Institute of Hematology and Transfusion Medicine Warsaw Poland; ^2^ Department of Soft Tissue/Bone Sarcoma and Melanoma Maria Sklodowska‐Curie National Research Institute of Oncology Warsaw Poland; ^3^ Department of Biostatistics Institute of Mother and Child Warsaw Poland; ^4^ Early Phase Clinical Trial Unit Maria Sklodowska‐Curie National Research Institute of Oncology Warsaw Poland

**Keywords:** BRAF mutations, Galectin‐1, immune escape, melanoma, PD‐L1

## Abstract

Although melanoma is considered one of the most immunogenic malignancies, spontaneous T‐cell responses to melanoma antigens are ineffective due to tumor cell‐intrinsic or microenvironment‐driven immune evasion mechanisms. For example, oncogenic BRAF V600E mutation in melanoma cells fosters tumor immune escape by modulating cell immunogenicity and microenvironment composition. BRAF inhibition has been shown to increase melanoma cell immunogenicity, but these effects are transient and long‐term responses are uncommon. For these reasons, we aimed to further characterize the role of BRAF‐V600E mutation in the modulation of PD‐L1, a known immunoregulatory molecule, and galectin‐1 (Gal‐1), a potent immunoregulatory lectin involved in melanoma immune privilege. We report herein that vemurafenib downregulates IFN‐γ‐induced PD‐L1 expression by interfering with STAT1 activity and by decreasing PD‐L1 protein translation. Surprisingly, melanoma cells exposed to vemurafenib expressed higher levels of Gal‐1. In coculture experiments, A375 melanoma cells pretreated with vemurafenib induced apoptosis of interacting Jurkat T cells, whereas genetic inhibition of Gal‐1 in these cells restored the viability of cocultured T lymphocytes, indicating that Gal‐1 contributes to tumor immune escape. Importantly, Gal‐1 plasma concentration increased in patients progressing on BRAF/MEK inhibitor treatment, but remained stable in responding patients. Taken together, these results suggest a two‐faceted nature of BRAF inhibition‐associated immunomodulatory effects: an early immunostimulatory activity, mediated at least in part by decreased PD‐L1 expression, and a delayed immunosuppressive effect associated with Gal‐1 induction. Importantly, our observations suggest that Gal‐1 might be utilized as a potential biomarker and a putative therapeutic target in melanoma patients.

## Graphical abstract

Melanoma is one of the most immunogenic malignancies, amenable for immunotherapeutic interventions. BRAF inhibition has been shown to increase melanoma cell immunogenicity, but these effects are transient and rapidly exhaustible. We demonstrate herein two facets of BRAF inhibition in melanoma cells: immunostimulatory activity due to decreased PD‐L1 transcription and translation, and immunosuppressive effect associated with Gal‐1 induction.

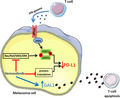

**Abbreviations**
CTCEA: common terminology criteria for adverse events
ELISA: Enzyme‐linked immunosorbent assay
ERK: extracellular signal‐regulated kinase
Gal‐1: galectin‐1
IFN‐γ: interferon γ
MAPKs: mitogen‐activated protein kinases
PD‐L1: programmed death‐ligand 1
PIM: proviral integration site for moloney murine leukemia virus kinase
RECIST: response evaluation criteria in solid tumors
STAT1: Signal transducer and activator of transcription 1

## 1 Introduction

Melanoma is a highly aggressive tumor of the skin, mucous membranes or uvea arising from pigment‐producing cells, melanocytes. Ultraviolet (UV) light exposure is a well‐established, major melanoma risk factor. Accordingly, melanoma cells exhibit a high prevalence of mutations caused by UV exposure, such as C>T mutations, pyrimidine dimers and other lesions repaired by transcription‐coupled nucleotide‐excision repair (Alexandrov *et al*., 2013). As nonsynonymous somatic mutations can create neoantigens—novel tumor‐specific protein epitopes that can be presented by MHC molecules and recognized by T cells as nonself, melanomas are considered highly immunogenic tumors. Nonetheless, spontaneous T‐cell responses to melanoma antigens are ineffective, due to melanoma‐intrinsic immune evasion mechanisms, such as the expression of PD‐L1/2 immune checkpoints (Keir *et al*., 2008; Pardoll, 2012). Clinically, immune checkpoint inhibitor (ICI)‐based therapies targeting PD‐1 and boosting neoantigen‐specific T‐cell responses have been shown to significantly alleviate melanoma’s immune escape mechanisms and improve patient survival (Curran *et al*., 2010; Dummer *et al*., 2015; Robert *et al*., 2015; Weber *et al*., 2015). In addition to PD‐1 checkpoint ligands, melanoma cells express galectin‐1, (LGALS‐1, Gal‐1), a potent immunomodulatory lectin that favors immune escape in multiple tumors (Cedeno‐Laurent *et al*., 2012; Juszczynski *et al*., 2007; Rubinstein *et al*., 2004; Tang *et al*., 2012). Gal‐1 inhibits T‐cell effector functions, blunts Th1 and Th17 responses, and skews the immune response toward a Th2‐type cytokine profile (Giordano *et al*., 2013; Juszczynski *et al*., 2007; Rabinovich and Toscano, 2009). In addition, this lectin instructs dendritic cells to become tolerogenic, induces alternatively activated ‘M2‐type’ macrophages, and favors the expansion of FoxP3 + regulatory T cells (Treg) and FoxP3‐, IL10 + type 1 regulatory (Tr1) cells (Cedeno‐Laurent *et al*., 2012; Ilarregui *et al*., 2009; Toscano *et al*., 2007), further limiting the magnitude of an effective immune response. Gal‐1 genetic inhibition with antisense oligonucleotides in a B16 murine melanoma model led to immune‐mediated rejection of the tumor (Rubinstein *et al*., 2004), suggesting that its expression in melanoma cells is an important mediator of tumor immune privilege.

The most common genetic abnormalities in melanoma are mutations of the MAPK (RAS‐BRAF/RAF‐MEK‐ERK) signal transduction pathway. BRAF mutations occur in 40–50% of melanoma patients, whereas NRAS mutations are identified in additional 15–20% (Chin, 2003; Chin *et al*., 2006; Eigentler *et al*, 2016; Flaherty *et al*., 2012; Gonzalez *et al*., 2013; Tsao *et al*., 2012). BRAF point mutations cluster in a specific region and usually result in a single phosphomimetic substitution in the kinase‐activation domain (V600E) that confers constitutive activation of BRAF and leads to uncontrolled, constitutive activity of downstream signaling pathways that stimulate cellular proliferation and increase tumor cell invasion, metastatic potential, and resistance to apoptosis (Chin, 2003; Pritchard and Hayward, 2013). In addition to tumor cell‐intrinsic consequences of oncogenic BRAF V600E mutation, it has been also implicated in fostering tumor’s immune escape, either by modulating cell immunogenicity or by modulating the microenvironment. For example, BRAF mutations impair antigen presentation in tumor cells (Bradley *et al*., 2015; Frederick *et al*., 2013), modulate expression of PD‐L1 immune checkpoint molecule (Zeng *et al*., 2016), and increase production of cytokines that reprogram tumor‐associated fibroblasts, macrophages, and dendritic cells to exhibit immunosuppressive properties (Khalili *et al*., 2012). In preclinical models and in retrospective analyses of patients undergoing BRAF inhibitor therapy, blockade of this pathway was linked to a more favorable microenvironment function, with an increased number of activated tumor‐infiltrating lymphocytes (Frederick *et al*., 2013). These findings appear particularly interesting in light of more recent studies, demonstrating that the number of tumor‐infiltrating lymphocytes (TILs) in the tumor tissue predicts better immunotherapy outcomes (Wong *et al*., 2019).

However, the increased infiltration of IFN‐γ‐producing activated T cells in the tumor tissue is associated with induction of PD‐L1 in the tumor cells. This effect, known as ‘adaptive immune resistance’, can limit the efficacy of immunotherapy (Ribas, 2015). Thus, understanding the mechanisms regulating PD‐L1 expression (and other potential immunoregulatory molecules) in melanoma patients is important to rationally manage the risk of clinical trial design and therapy failures. For these reasons, we aimed to characterize the role of BRAF‐V600E mutation in modulation of PD‐L1 and Gal‐1 expression and to further investigate potential immunoregulatory properties of BRAF inhibitors in melanoma. We report herein that vemurafenib downregulates IFN‐γ‐induced PD‐L1 expression in melanoma cells by interfering with the STAT1 activity and by decreasing PD‐L1 protein translation. Surprisingly, vemurafenib induced Gal‐1 expression in melanoma cells, leading to increased apoptosis of interacting T cells and potentially contributing to tumor immune privilege. Moreover, we have found that Gal‐1 plasma concentration increases in patients progressing on BRAF/MEK inhibitor treatment. Our results suggest that Gal‐1 might play an important role in tumor progression and could be a novel progression biomarker in melanoma patients.

## 2 Materials and methods

### 2.1 Cell lines and chemicals

Human melanoma cell lines A375, SK‐MEL5, and SK‐MEL28 were obtained from ATCC (Manassas, VA, USA) and maintained in DMEM (Lonza, Basel, Switzerland) supplemented with 10% (A375, SK‐MEL5) or 15% (SK‐MEL28) heat‐inactivated fetal bovine serum (Biovest, Riverside, MO, USA), 2 mm L‐glutamine, 10 mm HEPES, and 100 U·mL^−1^ of penicillin/streptomycin (Lonza). The Jurkat cell line was obtained from ATCC (Manassas, VA, USA) and maintained in RPMI 1640 medium (Lonza) supplemented with 10% heat‐inactivated fetal bovine serum (Biovest), 2 mm L‐glutamine, 10 mm HEPES, and 100 U·mL^−1^ of penicillin/streptomycin (Lonza). Cells were grown in a humidified atmosphere at 37 °C with 5% CO_2_. The 293T cell line was purchased from ATCC (Manassas, VA, USA) and maintained in DMEM supplemented with 10% FBS. Vemurafenib, cobimetinib, trametinib, and SGI‐1776 were purchased from Selleckchem (Houston, TX, USA) and dissolved in sterile dimethyl sulfoxide (DMSO). Interferon‐γ (IFN‐γ) was obtained from Thermo Fisher Scientific (Waltham, MA, USA).

### 2.2 Vectors and retroviral infection

The cDNA encoding PDL1‐Flag was generated in a two‐step PCR. At first, protein‐coding region without STOP codon of PD‐L1 was amplified using cDNA transcribed from A375 cell mRNA as a template and F1_PDL1_FLAG, R1_PDL1_FLAG primers (sequences provided in Table S1). Next, EcoRI and NgoMIV restriction sites, FLAG sequence (DYKDDDDK), and STOP codon were added in second PCR (primers: F2_PDL1_FLAG, R2_PDL1_FLAG). The gel‐purified PDL1‐FLAG fragment and pBabe‐puro were digested using EcoRI and NgoMIV (New England Biolabs, Ipswich, MA, USA) restriction enzymes and ligated to yield the pBabe‐PDL1_FLAG construct. The sequence was confirmed to be correct by Sanger sequencing. Previously described pSIREN‐RetroQ vector encoding Gal‐1‐specific shRNA (Gal1 shRNA) or scrambled control shRNA (Juszczynski *et al*., 2007) and pBabe‐MEK‐Q56P or pBabe‐MEK‐wt (Polak *et al*., 2016) were used.

The generation of retroviruses and infection was performed as described previously (Abramson *et al*., 2009; Gorniak and Juszczynski, 2018). Briefly, HEK‐293T cells were transfected with pKAT, VSV‐g, and a given retroviral plasmid using Lipofectamine 2000 reagent (Invitrogen, Carlsbad, CA, USA). Following 24‐h incubation at 37°C, the retroviral supernatant was collected, mixed with hexadimethrine bromide (8 μm final concentration; Sigma‐Aldrich, Saint Louis, MO, USA), and used to infect A375 cells when 60–80% confluency was reached. After infection, cells were subjected to antibiotic selection with 1 µg·mL^−1^ puromycin.

### 2.3 Luciferase assays

Previously described pGL3 luciferase vector (Promega, Madison, WI, USA) containing CD274 (PD‐L1) promoter sequence (Green *et al*., 2010) and pGL3 containing GAS sequence or ISRE sequence upstream of Firefly luciferase gene were used. For luciferase assay, the A375 cell line was grown to approximately 60–80% confluence on 6‐well plate and cotransfected with 1.0 μg/well of pGL3 luciferase construct and 0.5 μg/well pRL‐TK (Promega) using Lipofectamine 2000 reagent (Invitrogen). pRL‐TK vector encoding Renilla luciferase gene was used as a control reporter to allow for transfection efficiency normalization across different experimental conditions. After 24 h of incubation, cells were treated with 2.5μm vemurafenib, 0.1 μm trametinib, 0.1 μm cobimetinib, or the equivalent volume of dimethyl sulfoxide, and after next 2 h, IFN‐γ was added. After an additional 24 h of incubation, cells were lysed, and luciferase activities were determined by chemiluminescence assay with the use of the Dual‐Luciferase Assay Kit (Promega) and Tristar LB941 Berthold Luminometer as previously described (Sewastianik *et al*., 2016).

### 2.4 Immunoblotting and FACS

Cells were washed in PBS and lysed in RIPA buffer supplemented with protease and phosphatase inhibitor cocktail (Complete Protease Inhibitor Cocktail Tablets, PhosSTOP Phosphatase Inhibitor Cocktail Tablets, Roche, Basel, Switzerland) as described previously (Juszczynski *et al*., 2009). Proteins were size‐fractionated by sodium dodecyl sulfate (SDS)/PAGE and transferred to Immobilon PVDF membranes (Millipore, Burlington, MA, USA). Blots were incubated in blocking buffer (5% BSA, 0.1% Tween/Tris‐buffered saline TBS) at room temperature for 1 h and subsequently incubated with primary antibodies (listed in Table S2) diluted 1: 1000 in 5% BSA/TBST overnight at 4°C with rotation. After washing in TBST, blots were incubated with appropriate horseradish peroxidase (HRP)‐conjugated secondary antibodies at room temperature for 1 h, developed by ECL (PerkinElmer, Waltham, MA, USA), and visualized with G:Box image acquisition system (Syngene, Cambridge, UK). To reprobe with another antibody, blots were incubated in a stripping buffer at 50 °C for 30 min and analyzed as described above. Densitometric quantifications of band intensities were performed using imagej software (www.imagej.net).

The PD‐L1 and CD7 surface expressions were evaluated by flow cytometry as previously described (Green *et al*., 2010) with antibodies listed in Table S2. Cells were analyzed using FACSCanto flow cytometer (BD Biosciences, San Jose, CA, USA) and flowjo v.10 software (BD Bioscience).

### 2.5 Real‐time PCR

RNA was extracted using GeneMATRIX Universal RNA Purification Kit (EURx, Gdansk, Poland) and reverse‐transcribed with Transcriptor Universal cDNA Master (Roche). Gene expression levels were measured on CFX96 Real‐Time System (Bio‐Rad, Hercules, CA, USA) with the gene‐specific primers (sequences provided in Table S1) and SYBR Green PCR Master Mix (Life Technologies, Carlsbad, CA, USA). Obtained C_T_ values for PD‐L1, Gal‐1, PD‐L1_FLAG target genes, and a housekeeping control (glyceraldehyde 3‐phosphate dehydrogenase [GAPDH]) were used to calculate relative transcript abundance using ΔΔC_T_ method.

### 2.6 Metabolic protein labeling (Click‐iT) assay

To evaluate protein synthesis *de novo*, Click‐iT AHA (L‐azidohomoalanine) assays were used according to the manufacturer’s protocol. Briefly, A375 cells (60–80% confluent) were coincubated for 4 h with 50 µm Click‐iT AHA reagent and vemurafenib with/without IFN‐γ in a methionine‐free medium. Next, the cells were washed twice with PBS. For Alexa Fluor 488 staining of newly synthesized proteins, cells were fixed with 3.7% paraformaldehyde, blocked with 3% bovine serum albumin, and permeabilized with 0.5% Triton X‐100. In the next step, cells were labeled with Click‐iT reaction cocktail containing Alexa Fluor 488 alkyne and analyzed by flow cytometry. To assess the influence of vemurafenib on PD‐L1 protein synthesis, the cells were lysed with 1% sodium dodecyl sulfate and proteins labeled with AHA were conjugated with biotin alkyne. The lysates were precipitated twice, using chloroform and methanol protein precipitation method (Wessel and Flügge, 1984), to eliminate unbound biotin particles. Then, biotin‐labeled proteins were immunoprecipitated using Neutravidin Agarose Resin (Thermo Fisher Scientific) and separated by sodium dodecyl sulfate/polyacrylamide gel electrophoresis. PD‐L1 expression was subsequently assessed in biotinylated fraction by western blotting. GAPDH level in input samples served as a loading control.

### 2.7 Gal‐1‐induced T‐cell apoptosis assay

A375 control and A375_GAL1sh cells plated on 24‐well plate were treated with DMSO alone or vemurafenib (2.5 µm) for 24 h. Jurkat T cells were stimulated for 24 h using human T‐activator CD3/CD28 Dynabeads (Thermo Fisher Scientific), and 1 × 10^5^ of activated cells were mixed with pretreated A375 and A375_GAL1sh cells. After 24 h, apoptosis of Jurkat T cells was assessed in cocultures by flow cytometry. Cells were stained with APC‐CD7 antibody to discriminate T cells from A375 cells (T cells: CD7‐positive; A375 cells: CD7‐negative) and subsequently with Annexin V–fluorescein isothiocyanate (FITC) to evaluate apoptosis. Annexin V‐FITC staining was performed according to the manufacturer’s instructions (Annexin V‐FITC Apoptosis Detection Kit I, BD Biosciences). Apoptotic cells were evaluated in the CD7‐positive population using BD FACSCanto flow cytometer (BD Biosciences) and analyzed by flowjo v.10 software (BD Bioscience).

### 2.8 Patients, plasma samples, and ELISA

Before patient enrollment, the Bioethical Committee issued a positive decision about this pilot study, and the signed informed consent from all patients was obtained. Nine enrolled patients had been diagnosed with metastatic melanoma with BRAF mutation. All patients received a combination of BRAF + MEK inhibitors, no grade 3–5 toxicity acc. CTCEA were observed during treatment. The patients’ sera were collected before the first administration of BRAF + MEK inhibitors and every 1–6 months from initiation of treatment at the time of radiological and clinical assessment. Radiological assessment was done according to RECIST 1.1. Patients’ characteristics and therapy response status are presented in Table S3. Soluble Gal‐1 in patients’ plasma was quantified using a commercial ELISA kit (Human Galectin‐1 Quantikine ELISA Kit; R&D systems, Minneapolis, MN, USA), according to the manufacturer’s protocol. Serum was diluted ten times, and all samples were analyzed in duplicates.

### 2.9 Statistical analyses

Differences between variables were performed with Student’s *t*‐test or one‐way ANOVA, with the Tukey HSD test that corrects for family‐wise error‐rate in multiple comparisons. *P* values < 0.05 were considered statistically significant: **** for *p < *0.0001; *** for *p < *0.001; ** for *p < *0.01; and * for *p < *0.05. All calculations were performed using graphpad prism 6 software (San Diego, CA, USA).

## 3 Results

### 3.1 Vemurafenib downregulates interferon‐γ‐induced PD‐L1 expression in melanoma cells by interfering with the STAT1 activity

**Fig. 1:**
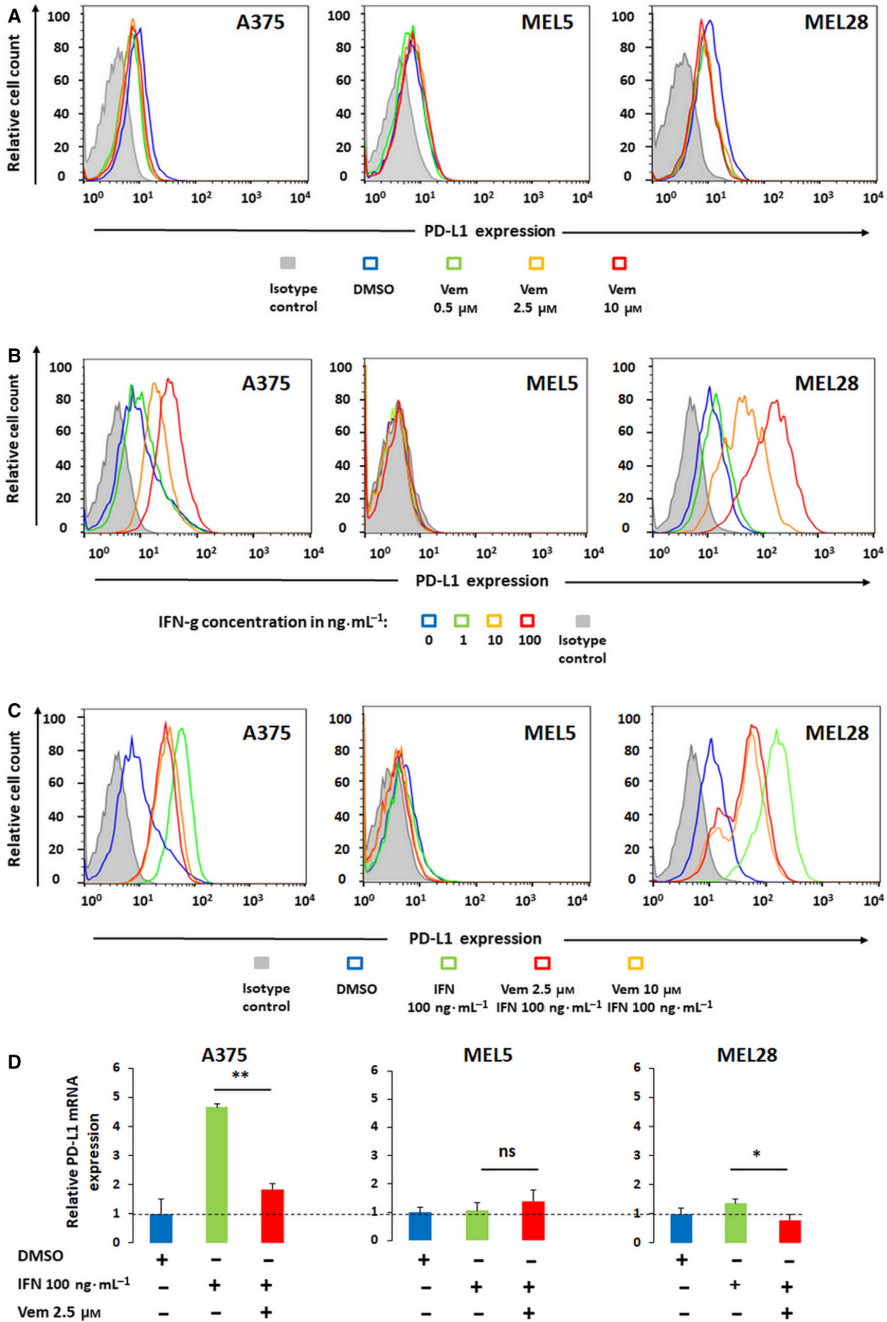
Vemurafenib curtails IFN‐γ‐induced PD‐L1 expression in melanoma cells. (A) PD‐L1 expression in A375, MEL5, and MEL28 melanoma cells at baseline and after 24 h incubation with vemurafenib. PD‐L1 expression was assessed by flow cytometry. (B) IFN‐γ‐inducible expression of PD‐L1 in melanoma cell lines. Cells were stimulated with IFN‐γ for 24 h, and PD‐L1 expression was assessed as in panel A. (C) Vemurafenib decreases IFN‐γ‐inducible PD‐L1 surface expression in melanoma cells. Cells were pretreated with vemurafenib for 2h and then incubated with IFN‐γ for 24h. PD‐L1 expression was assessed as in panel A. (D) Vemurafenib decreases IFN‐γ‐inducible CD274 (PD‐L1) transcript levels in melanoma cells. Cells were pretreated with the inhibitor for 2h and then incubated with IFN‐γ for additional 24h. Thereafter, relative *CD274* (PD‐L1) mRNA expression was assessed by real‐time PCR. GAPDH was used as a reference gene. The data from two independent experiments are presented. Differences in expression were assessed by the *t*‐test; asterisks indicate *p* values: ** for *p < *0.01; * for *p < *0.05. Error bars represent the standard deviations (SDs).

**Fig. 2:**
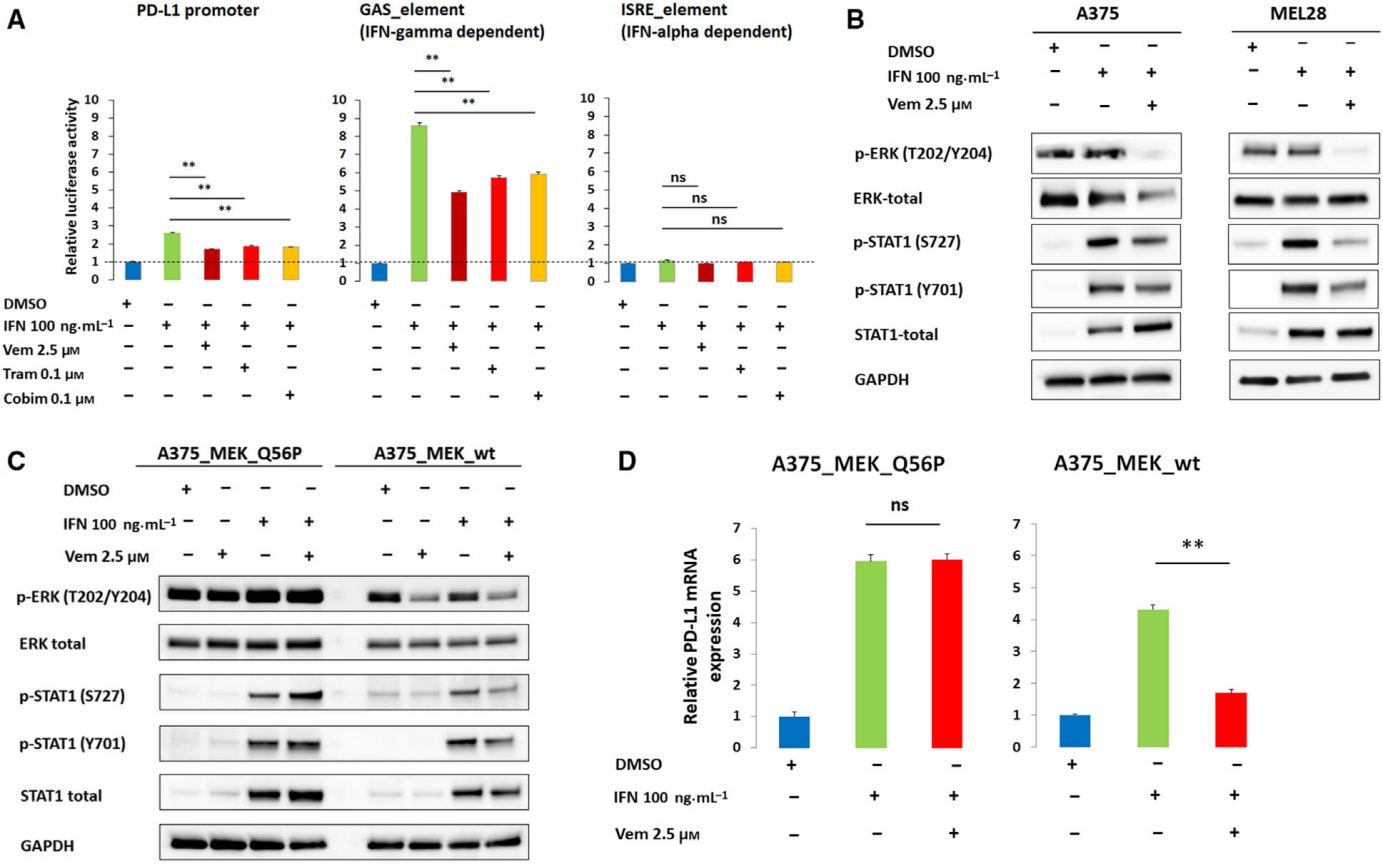
Vemurafenib reduces IFN‐γ‐induced, STAT1‐mediated *CD274* (PD‐L1) gene transactivation. (A) Vemurafenib reduces transcription from IFN‐γ‐inducible promoters. A375 cells were transfected with *CD274* (PD‐L1) promoter‐, GAS (gamma‐activated site) promoter‐, or ISRE (IFN‐α‐stimulated response element) promoter‐containing luciferase constructs. Luciferase activities were determined after 24 h incubation with IFN‐γ alone or in combination with vemurafenib, cobimetinib, or trametinib. The data from three independent experiments are presented. Differences in expression were assessed using one‐way ANOVA and Tukey HSD test; ** indicates *p < *0.01; * for *p < *0.05. Error bars represent the SD. (B) Vemurafenib reduces phospho‐ERK (T202/Y204) and STAT1‐activating phosphorylations. Pospho‐STAT1 (Y701 and S727) and phospho‐ERK levels were assessed after 24 h incubation with IFN‐γ alone and in combination with vemurafenib. GAPDH served as a loading control. Band intensities were quantitated by densitometry and are provided in Table S4a. (C) Vemurafenib does not modulate STAT1‐activating phosphorylations in cells with constitutive MEK activity. A375 cells were transduced with constitutively active MEK mutant (MEK_Q56P) or MEK wild‐type (MEK_wt) and treated with IFN‐γ alone or in combination with vemurafenib for 24h. Phospho‐ERK (T202/Y204) and phospho‐STAT1 (Y701 and S727) levels were assessed by immunoblotting. GAPDH served as a loading control. Band intensities were quantitated by densitometry and are provided in Table S4b. (D) Vemurafenib does not change PD‐L1 transcript levels in cells with constitutive MEK (MEK_Q56P) activity. MEK_Q56P and MEK_wt‐transduced cells were pretreated with the inhibitor for 2 h and then incubated with IFN‐γ for 24 h. Thereafter, relative PD‐L1 mRNA levels were assessed by real‐time PCR. GAPDH was used as a reference gene. The data from two independent experiments are presented. Differences in expression were assessed by t‐test; asterisks indicate *P* values: ** for *p < *0.01; * for *p < *0.05. Error bars represent the SD.

We first analyzed the baseline expression of PD‐L1 and the influence of vemurafenib on the expression of this molecule in three BRAF‐mutated human melanoma cell lines, A375, MEL5, and MEL28 using flow cytometry. In all three cell lines, the baseline level of PD‐L1 was low and was not markedly changed by vemurafenib (Fig. 1A). In immunogenic tumors, such as melanoma, the interferons released by tumor‐infiltrating T cells trigger the inducible expression of PD‐L1 by cancer cells, thereby inhibiting the antitumor immune response in a process known as ‘adaptive immune resistance’ (Ribas, 2015). Thus, we examined PD‐L1 surface expression in melanoma cell lines after IFN‐γ stimulation and its changes caused by vemurafenib. IFN‐γ massively induced PD‐L1 transcript levels and surface expression in a dose‐dependent manner in A375 and MEL28 cell lines, but not in MEL5 cells (Fig. 1B). Vemurafenib markedly decreased IFN‐γ‐induced PD‐L1 transcript and protein levels in these two cell lines (Fig. 1C,D). In addition, two MEK inhibitors, cobimetinib and trametinib, exhibited similar effects, indicating that BRAF‐MEK pathway blockade specifically dampens IFN‐γ signaling (Fig. S1). To further confirm these observations, we generated a reporter A375 cell line with luciferase gene downstream of the PD‐L1 promoter, the IFN‐γ‐responsive GAS (gamma‐activated site) or IFN‐α‐inducible ISRE (IFN‐α‐stimulated response element) synthetic promoters. IFN‐γ increased relative luciferase activity in cells transfected with PD‐L1 promoter‐ or GAS promoter‐containing constructs. In line with previous observations, vemurafenib significantly decreased IFN‐γ‐induced luciferase activity (Fig. 2A). Taken together, these results indicate that transcriptional PD‐L1 induction in melanoma cells is driven by IFN‐γ and can be specifically decreased in BRAF‐mutated cells by inhibition of this kinase.

Since immediate IFN‐γ downstream signaling effectors are STAT1 homodimers, we determined the changes in the phosphorylation status of STAT1 Y701 and S727, the two residues phosphorylated upon IFN‐γ receptor engagement. Tyrosine 701 is phosphorylated by JAK1/2 and is required for STAT1 dimerization, whereas S727 can be phosphorylated by several different kinases, including ERK1, and is critical to convey full transactivation potential to this transcription factor (Li *et al*., 2010). As ERK1 operates downstream of BRAF and MEK, we first confirmed that ERK1 activity decreases following incubation with vemurafenib (Fig. 2B, Fig. S2). Next, we assessed STAT1 phosphorylation at Y701 and S727 after incubation with IFN‐γ alone or in combination with vemurafenib in A375 and MEL28 cells. IFN‐γ led to increased STAT1 phosphorylation at Y701 and S727 in analyzed cell lines. As predicted, vemurafenib markedly decreased STAT S727 phosphorylation, and modestly decreased Y701 phospho‐levels (Fig. 2B). These results suggest that the constitutive activity of BRAF V600E fosters IFN‐γ signaling and PD‐L1 transcriptional activation by augmenting STAT1 S727 phosphorylation.

To confirm the role of the BRAF/MEK/ERK pathway in the modulation of the IFN‐γ pathway, we overexpressed the constitutively active form of MEK1 kinase (MEK‐Q56P) in A375 cells. MEK‐Q56P mutation, identified in melanoma patients, is located in the proximity of regulatory helix A and leads to constitutive kinase activity (Emery *et al*., 2009). As MEK is situated downstream of BRAF, we expected that MEK‐Q56P would substitute the signal terminated by vemurafenib. Indeed, comparing with MEK wild‐type, cells transduced with this MEK1 mutant did not exhibit changes in p‐ERK, p‐STAT(S727), and p‐STAT(Y701) after vemurafenib treatment (Fig. 2C). Consistent with these observations, vemurafenib did not decrease IFN‐γ‐induced PD‐L1 transcript abundance in MEK‐Q56P‐transduced cells (Fig. 2D).

### 3.2 Vemurafenib inhibits PD‐L1 protein synthesis

**Fig. 3:**
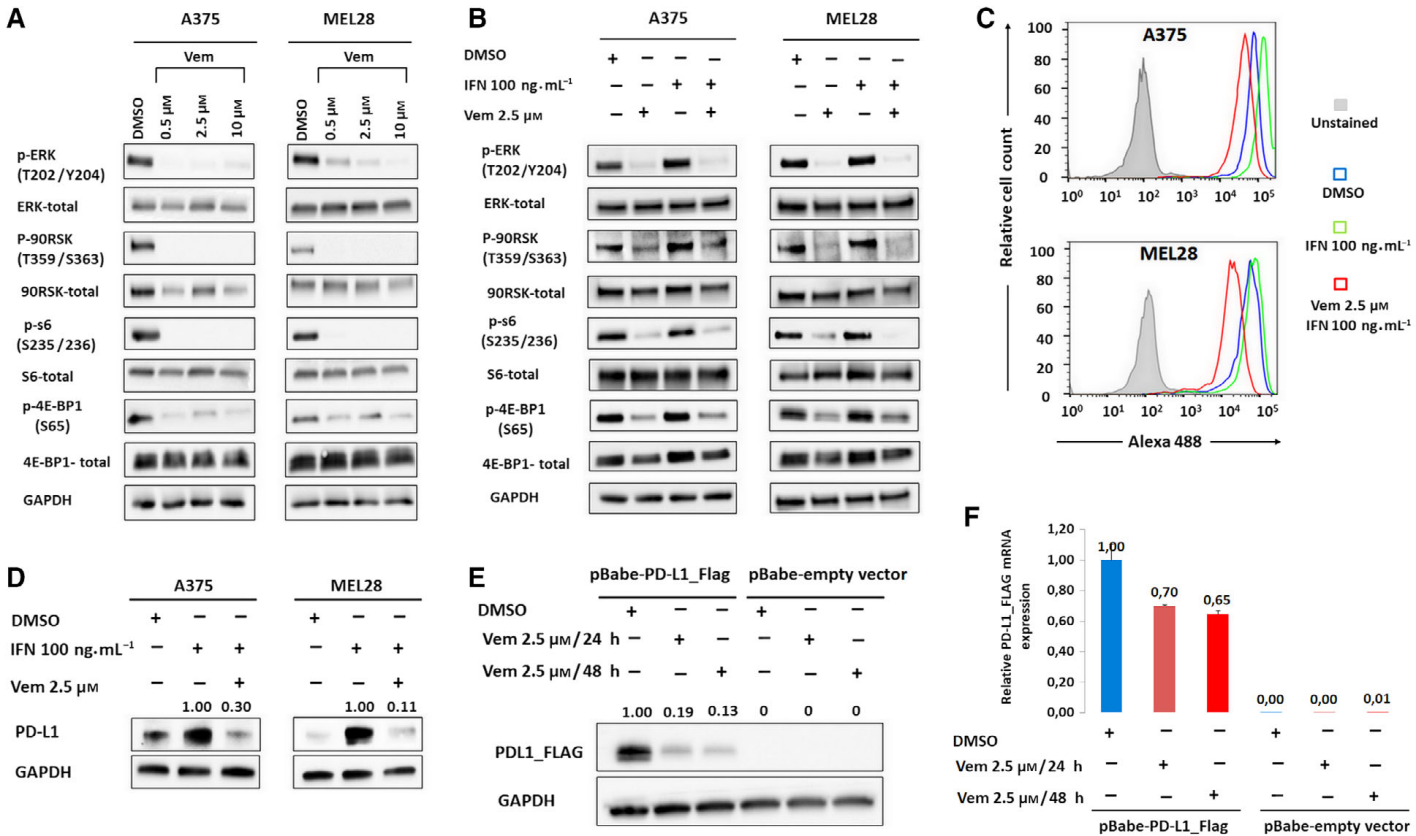
Vemurafenib inhibits protein translation. (A) Changes in phosphorylation of proteins regulating protein translation after 24 h incubation with vemurafenib in A375 and MEL28 melanoma cell lines, analyzed by western blot. GAPDH served as a loading control. Band intensities were quantitated by densitometry and are provided in Table S4c. (B) Changes in phosphorylation of proteins regulating protein translation after 24 h incubation with IFN‐γ alone and in combination with vemurafenib. A375 and MEL28 melanoma cells were pretreated with the inhibitor for 2 h and then incubated with IFN‐γ for 24h. GAPDH served as a loading control. Band intensities were quantitated by densitometry and are provided in Table S4d. (C) Global *de novo* protein synthesis after 24 h incubation with IFN‐γ alone and in combination with vemurafenib. A375 and MEL28 cells were pretreated with inhibitor for 2 h and then incubated with IFN‐γ for 24 h. Newly synthesized proteins were labeled with methionine analogue L‐azidohomoalanine, conjugated with Alexa Fluor 488‐alkyne, and analyzed by flow cytometry. (D) *D* *e novo* PD‐L1 protein synthesis in melanoma cells stimulated with IFN‐γ alone or pretreated with vemurafenib. Proteins labeled with L‐azidohomoalanine were conjugated with biotin–alkyne, precipitated using avidin‐conjugated beads, and immunoblotted with α‐PD‐L1 antibody. (E) Vemurafenib decreases abundance of FLAG‐PD‐L1 protein expressed from IFN‐unresponsive (LTR) promoter. A375 cells were transfected with pBabe‐PD‐L1_Flag vector and treated with vemurafenib for 24 h, and FLAG‐tagged PD‐L1 protein abundance was assessed by western blot and quantified using band densitometry; GAPDH served as a loading control. (F) Relative PD‐L1_FLAG transcript levels in A375 cells were transduced with pBabe‐PD‐L1_Flag and treated with vemurafenib as in panel E. Transcript abundance was measured by real‐time PCR. GAPDH was used as a reference gene. The data from two independent experiments are presented. Error bars represent the SD.

Thereafter, we sought for additional vemurafenib‐modulated mechanisms that might be responsible for PD‐L1 protein expression. To this end, we studied vemurafenib’s effect on protein translation by assessing activity of proteins involved in translation regulation: translation repressor protein 4E‐BP1 (Pause *et al*., 1994), S6 ribosomal protein (Peterson and Schreiber, 1998), and ribosomal S6 kinases (p90RSK) that are activated via coordinated phosphorylation by MAPKs (Gwin *et al*., 2011; Romeo *et al*., 2013). Exposure to increasing vemurafenib concentrations led to a dramatic decrease in phosphorylation of all analyzed proteins (Fig. 3A). Similar effect of vemurafenib was observed in the presence of IFN‐γ (Fig. 3B). To show the direct impact of vemurafenib on protein translation, we utilized azide‐alkyne cycloaddition (‘Click‐iT’) chemistry to label newly synthesized proteins. This analysis revealed that vemurafenib markedly dampened global protein synthesis (Fig. 3C). To specifically assess the vemurafenib’s effect on PD‐L1 translation, we biotinylated newly synthesized proteins using Click‐iT reaction, pulled down biotin‐labeled proteins using avidin‐coated beads and immunoblotted obtained protein concentrate with an anti‐PD‐L1 antibody. This experiment indicated that vemurafenib decreased PD‐L1 translation (Fig. 3D).

To rule out the possibility that decreased translation of PD‐L1 results from the decreased expression of its transcript, we transduced A375 cells with a retroviral vector construct containing the FLAG‐tagged PD‐L1 gene, in which transcription is independent of the physiological regulatory region and driven only by the LTR promoter. Western blot analysis performed on this model showed that vemurafenib treatment caused massive decrease (81–87%) in PD‐L1_FLAG protein abundance (Fig. 3E), whereas PD‐L1_FLAG transcript levels decreased only by 30%‐35% (Fig. 3F). Taken together, these results confirm that vemurafenib decreases PD‐L1 translation.

### 3.3 Vemurafenib increases expression of immunoregulatory protein galectin‐1

**Fig. 4:**
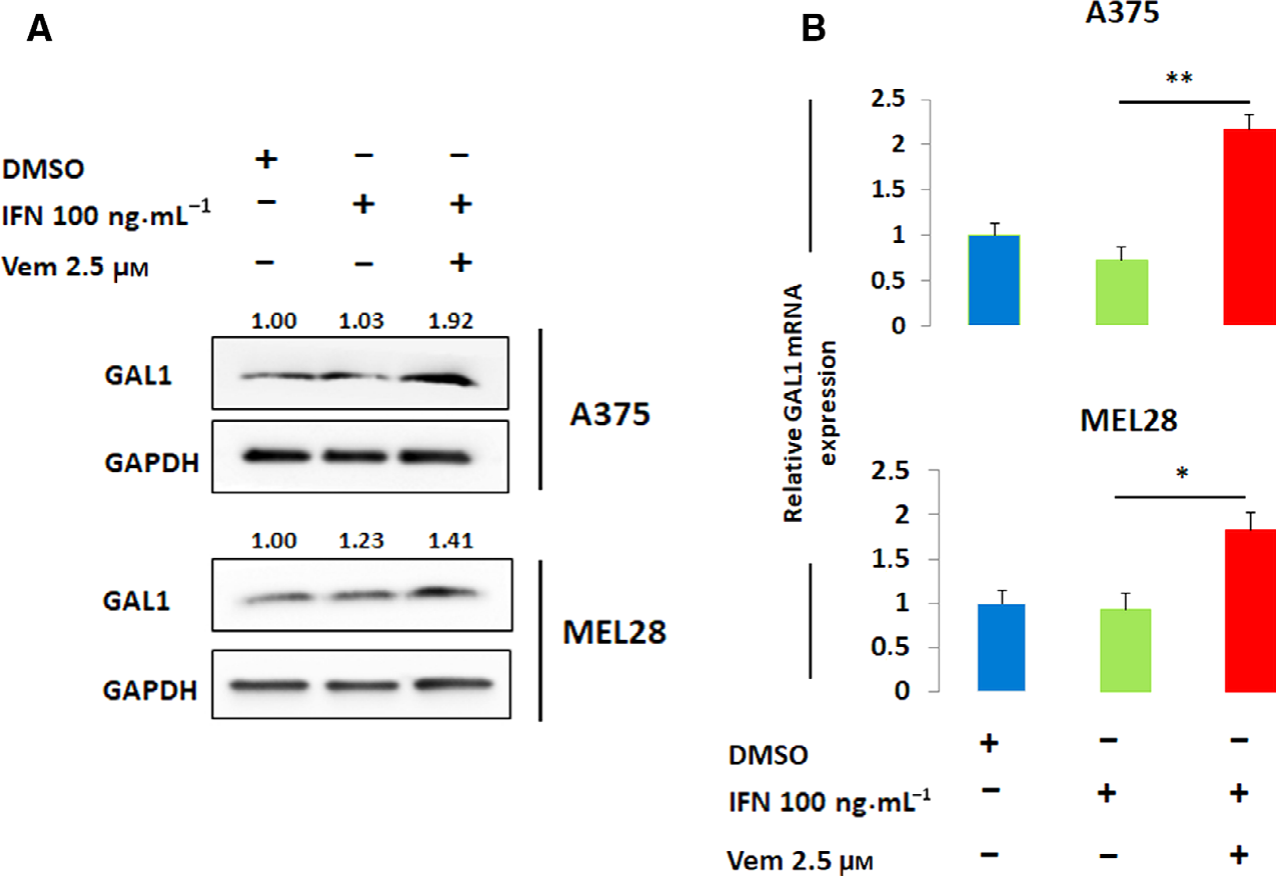
Vemurafenib increases expression of an immunoregulatory protein, galectin‐1. (A) Gal‐1 protein expression after 24 h incubation with IFN‐γ alone and in combination with vemurafenib. A375 and MEL28 cells were pretreated with inhibitor for 2 h and then incubated with IFN‐γ for 24 h. Gal‐1 abundance was assessed by western blot and quantified using band densitometry. GAPDH served as a loading control. (B) Relative Gal‐1 transcript level in A375 and MEL28 after 24 h incubation with IFN‐γ alone and in combination with vemurafenib. mRNA levels were determined using real‐time PCR. GAPDH was used as a reference gene. The data from two independent experiments are presented. Differences in expression were assessed by *t*‐test; asterisks indicate *P* values: ** for *p < *0.01; * for *p < *0.05. Error bars represent the SD.

Having demonstrated the downregulation of surface PD‐L1 expression by BRAF inhibition, we hypothesized that vemurafenib should trigger increased activation of T cells interacting with vemurafenib‐pretreated melanoma cells. Surprisingly, the expression of T‐cell activation markers (CD25 and CD69) on Jurkat T cells cocultured with IFN‐γ or IFN‐γ + vemurafenib‐pretreated melanoma cells remained unchanged (Fig. S3), indicating that either the magnitude of PD‐L1 downregulation is insufficient to alleviate T‐cell exhaustion or the decreased expression of PDL1 on melanoma cells treated with vemurafenib is compensated by an increased level of a different checkpoint/immunoregulatory molecule. Unlike other checkpoints that induce T‐cell exhaustion and cooperate with PD‐L1, Gal‐1 exhibits different mechanisms of activity, inducing cell death specifically in activated T cells (Juszczynski *et al*., 2007; Long *et al*., 2018; Rubinstein *et al*., 2004; Sakuishi *et al*., 2010). To determine the influence of vemurafenib on Gal‐1 expression, A375 and MEL28 cells were incubated with vemurafenib alone or in combination with IFN‐γ and Gal‐1 transcript and protein abundance were assessed. Consistent with the hypothesis, vemurafenib treatment markedly increased Gal‐1 expression in A375 and MEL28 melanoma cells (Fig. S4, Fig. 4A,B).

### 3.4 Vemurafenib‐induced galectin‐1 in melanoma cells increases apoptosis in interacting T cells

**Fig. 5:**
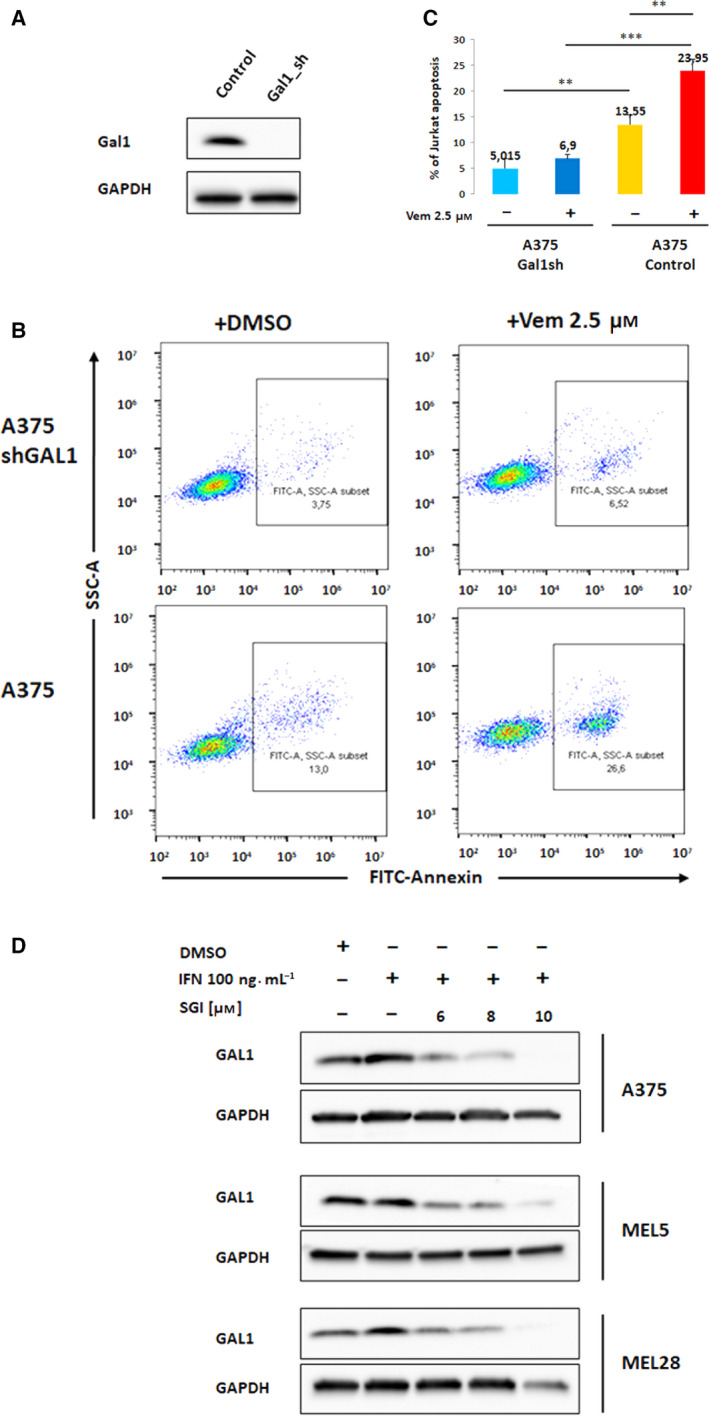
Vemurafenib‐induced Gal‐1 in melanoma cells increases apoptosis of interacting T cells. (A) Decreased Gal‐1 protein expression in A375 cells transduced with Gal‐1‐specific short hairpin RNA (Gal1_sh) or a scrambled control shRNA. (B and C) Viability of Jurkat T cells cocultured with Gal1 shRNA or a scrambled control shRNA A375 cells in the presence or absence of vemurafenib (24 h incubation), analyzed by Annexin V staining. Graphs in panel C represent averaged data from 2 independent experiments. Differences in apoptosis between the samples were assessed by the *t*‐test; asterisks indicate p values: *** for *p < *0.001; ** for *p < *0.01. Error bars represent the SD. (D) PIM kinase inhibition decreases Gal‐1 protein expression. A375, MEL5, and MEL28 cells were treated with SGI‐1776 pan‐PIM inhibitor for 2 h and with IFN‐γ for additional 24 h. Gal‐1 abundance was assessed by western blot. GAPDH served as a loading control.

Given the upregulation of Gal‐1 following BRAF inhibition in melanoma cell lines, we hypothesized that increased expression/secretion of Gal‐1 would facilitate the deletion of tumor‐interacting T cells. To test this hypothesis, we first generated stable A375 transfectants expressing Gal‐1‐specific short hairpin RNA (Gal‐1_sh) or a scrambled control shRNA (Fig. 5A). Thereafter, Gal‐1_shRNA or control A375 monolayers were pretreated with vemurafenib (2.5 μm) or DMSO for 24h to induce Gal‐1 expression. Importantly, A375 cells remained viable under these conditions and thus could be further utilized in subsequent experiments. After DMSO/vemurafenib preincubation, A375 cells were overlaid with CD3/CD28‐activated Jurkat T cells and cocultured for additional 24h. Consistent with the proapoptotic function of Gal‐1, the percentage of apoptotic T cells coincubated with control (Gal‐1‐expressing) DMSO‐treated A375 cells was higher than after coincubation with Gal‐1‐depleted cells (13% vs 5%, respectively; Fig. 5B left upper and lower panels and Fig. 5C). The percentage of apoptotic T cells markedly increased (from 13% to 24%) after coculture with vemurafenib‐pretreated, Gal‐1‐sufficient, control A375 cells, whereas apoptosis of T cells cocultured with vemurafenib‐pretreated, Gal‐1‐depleted A375 cells remained low (6,5% of Annexin V‐positive cells; Fig. 5B right upper and lower panels and Fig. 5C). These studies directly demonstrate that the endogenous and vemurafenib‐induced Gal‐1 decreases the viability of activated T cells. More importantly, blocking melanoma‐specific expression of Gal‐1 restores viability of these T cells.

We have recently demonstrated that PIM kinase inhibition decreases Gal‐1 expression in classical Hodgkin lymphoma (Szydłowski *et al*., 2017). Since PIM kinases are expressed by human melanoma tissue (Shannan *et al*., 2016), we investigated whether a pan‐PIM kinase inhibitor, SGI‐1776, decreases Gal‐1 protein expression in melanoma cell lines. Incubation of melanoma cells with SGI‐1776 markedly and in a dose‐dependent manner reduced Gal‐1 protein abundance (Fig. 5D), indicating that PIM inhibition is a potential immunomodulatory strategy in this disease.

### 3.5 Galectin‐1 plasma concentration increases in patients progressing on BRAF/MEK inhibitor treatment

**Fig. 6:**
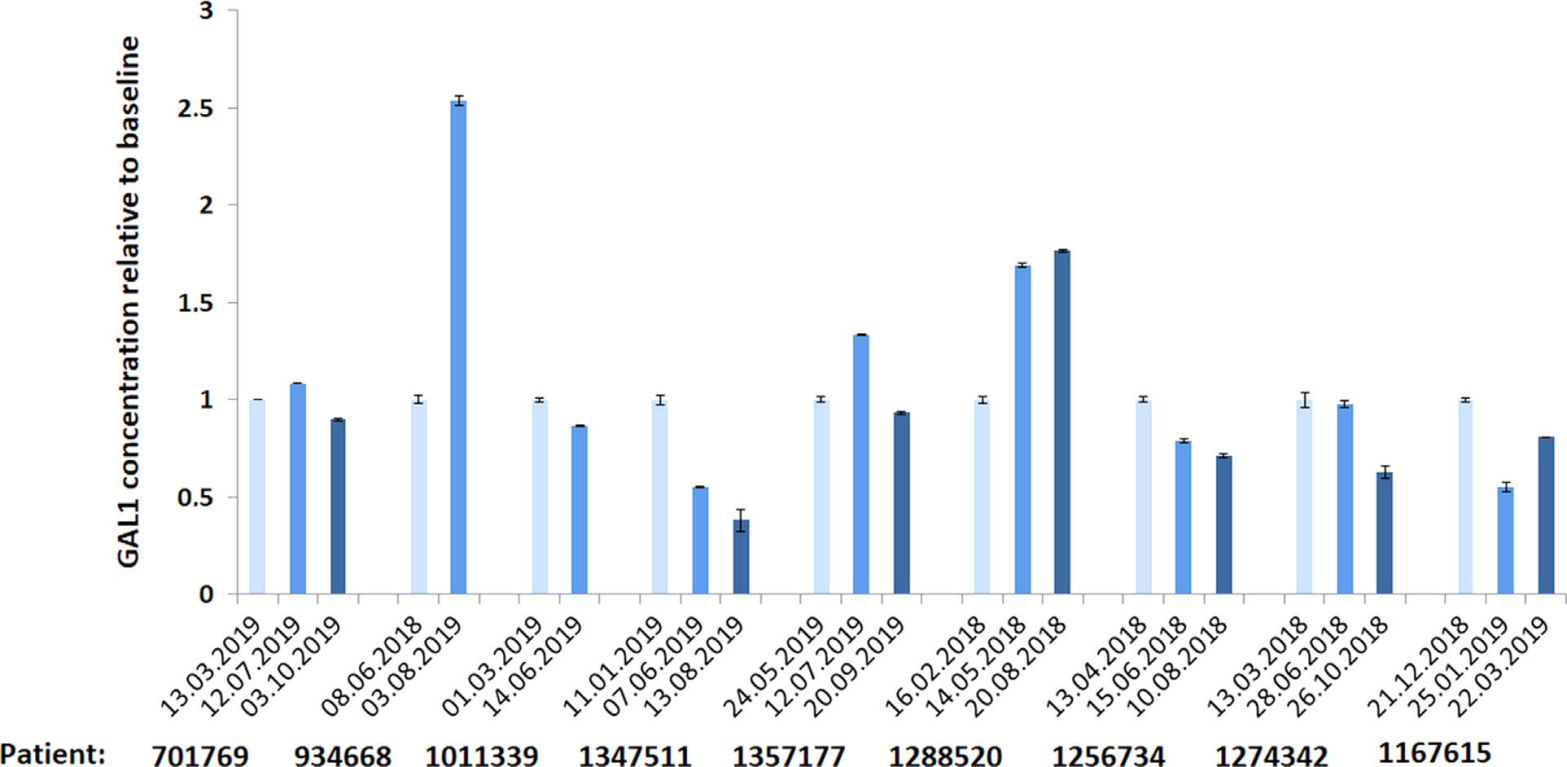
Gal‐1 plasma concentration in patients treated with BRAF + MEK inhibitors, determined using ELISA kit. Patient’s radiological responses are detailed in Table S3. Marked increase in Gal‐1 plasma levels was noted only in a progressing patient (#934668), who eventually deceased. In a patient #1288520, in the first measurement, Gal‐1 plasma concentration moderately increased, but stabilized thereafter. In the corresponding clinical assessment, patient had stable disease. In remaining responding patients, Gal‐1 plasma levels remained stable. The data from two technical replicates are presented. Error bars represent the SD.

Given the upregulation of Gal‐1 following BRAF inhibition in melanoma cell lines and T‐cell apoptosis‐inducing activity of this protein, we hypothesized that increased expression/secretion of Gal‐1 in BRAF inhibitor‐treated patients would facilitate deletion of tumor‐infiltrating T cells and might represent a mechanism of disease immune escape and progression. To test this hypothesis, we longitudinally evaluated Gal‐1 plasma concentrations at subsequent time points during the course of BRAF/MEK inhibitor therapy (first measurement before drug administration) in 9 metastatic melanoma patients (Table S3). Since Gal‐1 is a soluble protein, its concentrations were determined using ELISA. Gal‐1 markedly increased (>2‐fold change) only in a patient progressing on treatment (patient #934668). In the remaining patients achieving at least SD, Gal‐1 plasma concentrations remained stable over the course of follow‐up (Fig. 6). These results suggest that Gal‐1 might play an important role in tumor progression and could be a novel progression biomarker in melanoma patients.

## 4 Discussion

Due to high mutational load and neoantigen formation, melanoma is considered one of the most immunogenic malignancies (Erdag *et al*., 2012; Oble *et al*., 2009). However, most tumors develop a strong arsenal of immunomodulatory mechanisms that dampen the host immune response. BRAF mutations have been shown to contribute to this effect due to distinct tumor‐intrinsic and microenvironment‐specific mechanisms. Consistent with this, BRAF inhibitor treatment increases antigen presentation and favors cytotoxic T‐cell infiltration. However, in patients treated with BRAF inhibitors, increasing T‐cell infiltration is paralleled by the rising expression of PD‐L1. Mechanistically, this effect is mediated by JAK/STAT signaling pathway triggered by IFN‐γ produced by activated T cells. This mechanism, known as ‘adaptive immune resistance’, might mitigate the long‐term benefits of targeted therapy with BRAF inhibitors (Flaherty *et al*., 2012; Chin *et al*., 2006).

In this study, we demonstrate that in melanoma cells, BRAF inhibition curtails IFN‐γ‐induced PD‐L1 expression through two complementary mechanisms: decreased STAT1 activity and attenuation of protein translation. These results are consistent with other studies, indicating that MAPK inhibition in melanoma cells decreases PD‐L1 expression via modulation of c‐JUN/AP1 activity (Jiang *et al*., 2013). In addition, IFN‐γ‐dependent PD‐L1 expression can be modulated by NF‐κB; thus, inhibition of IKK downstream of BRAF prevents degradation of IκB subunits, retains NF‐κB in the cytoplasm, and blocks PD‐L1 expression (Gowrishankar *et al*., 2015; Hartman *et al*., 2017). Together, these mechanisms limit the magnitude of PD‐L1 induction caused by IFN‐γ produced by T cells infiltrating the tumor. In our observations, the surface PD‐L1 abundance induced by IFN‐γ was by an order of magnitude lower when the cells were incubated in the presence of vemurafenib. From the immunotherapy standpoint, curtailed PD‐L1 expression appears to be a beneficial effect and speaks for simultaneous, rather than sequential, use of targeted pathway inhibitors and anti‐PD‐1 antibodies.

Nonetheless, in patients treated with BRAF inhibitors alone, the duration of clinical responses to therapy is relatively short due to evolving resistance. Upon progression, BRAF inhibitor‐related, positive immunoregulatory effects are cleared, suggesting that BRAF inhibitor resistance is associated with an immunosuppressed tumor microenvironment, potentially contributing to a lack of response to subsequent anti‐PD‐1 therapy (Frederick *et al*., 2013). For example, some recent studies indicate that in progressing melanoma patients, prior BRAF inhibitor therapy reduced the efficacy of subsequent ICI therapy with nivolumab, suggesting that extended BRAF inhibitor treatment either impairs immune‐competent cells in the microenvironment or selects tumor cells capable of escaping the attack (Johnson *et al*., 2017; Simeone *et al*., 2017). In addition, increased tumor T‐cell infiltration in BRAF inhibitor‐treated patients disappears in biopsies collected after 15 days from treatment initiation, suggesting that tumor cells develop a ‘counter‐strike’ strategy to delete T cells (Deken *et al*., 2016). Taken together, the immunoregulatory effects of BRAF inhibitor appear to be transient, exhaustible, or compensated by other mechanisms. Consistent with these clinical observations, we noted that BRAF inhibitor‐exposed cells increase the expression of Gal‐1, a potent immunoregulatory lectin capable of reprogramming the microenvironment and deleting activated T cells. Increased expression of this lectin was also observed in the plasma of BRAF/MEK inhibitor‐treated, progressing melanoma patients. Vemurafenib‐induced Gal‐1 in melanoma cells was sufficient to initiate apoptotic cell death in interacting Jurkat T cells. Importantly, genetic inhibition of Gal‐1 by RNA interference almost completely blocked apoptosis of T cells in cocultures with vemurafenib‐pretreated tumor cells, confirming that this effect is specifically mediated by Gal‐1. Given broad and pleiotropic Gal‐1 functions, the expression of this protein might be a likely explanation to several additional effects observed in the tumor microenvironment following BRAF inhibition. In a BRAF V600E mutant syngeneic SM1 melanoma model, BRAF inhibition was associated with increased regulatory T cells (Treg), macrophage, and myeloid‐derived suppressor cell (MDSC) infiltrates (Hu‐Lieskovan *et al*., 2015). Since Gal‐1 favors the expansion of these cell populations (Juszczynski *et al*., 2007; Rubinstein *et al*., 2004), increased infiltrates of these cells in BRAF inhibitor‐treated melanomas might be driven, at least partially, by Gal‐1 overexpression.

However, the molecular mechanism leading to Gal‐1 induction in vemurafenib‐responding melanoma patients remains unclear. Given increased plasma Gal‐1 concentration in a progressing patient, it is more likely to be an indirect/compensatory effect, rather than a direct consequence of BRAF‐MEK‐ERK pathway inhibition. Importantly, Gal‐1 is regulated by multiple pathways (e.g., NFκB and AP‐1 transcription factors, hypoxia, redox balance), which are deregulated in progressing treatment‐refractory patients (Juszczynski *et al*., 2007; Toscano *et al*., 2011; Xu‐Yun *et al*., 2010). Moreover, apoptosing tumor cells in patients responding to BRAF inhibitor will not be able to produce Gal‐1, whereas in progressing patients, increasing tumor volume/mass will likely produce more lectin. Regardless of the underlying molecular mechanism, induction of Gal‐1 in BRAF inhibitor‐treated, progressing melanoma patients is an important observation from the translational standpoint. First, Gal‐1 expression has been shown to be a valuable diagnostic and prognostic biomarker in multiple malignancies (Kamper *et al*., 2011; Juszczynski *et al*., 2010; Ouyang *et al*., 2013; Rodig *et al*., 2008). Given the simplicity of Gal‐1 plasma concentration measurements, this approach can be implemented in diagnostic laboratories as a biomarker identifying progressing patients. Prospective studies that will address this hypothesis are currently ongoing. Second, as Gal‐1 is a potentially targetable protein (Ouyang *et al*., 2011; Rubinstein *et al*., 2004), its inhibition might alleviate the detrimental effects in the tumor microenvironment and impede tumor immune escape. However, since direct Gal‐1 inhibitors are not available for clinical use, other indirect approaches might be an attractive alternative. We have recently shown that PIM kinase inhibitors decrease the Hodgkin lymphoma‐specific expression of Gal‐1 (Szydłowski *et al*., 2017). Herein, we recapitulate this result in melanoma cells, demonstrating that a pan‐PIM inhibitor SGI‐1776 decreases Gal‐1 abundance. Given the ongoing clinical development of pan‐PIM inhibitors, this observation is particularly interesting. It is tempting to hypothesize that PIM inhibitors might prevent the adverse microenvironmental effects of BRAF inhibitors and favor the host’s immune tumor control.

## 5 Conclusions

Taken together, studies presented herein suggest two‐faceted nature of BRAF inhibition‐associated immunomodulatory effects: early immunostimulatory activity, mediated at least in part by decreased PD‐L1 expression, and a delayed immunosuppressive effect associated with Gal‐1 induction. Importantly, our observations suggest that Gal‐1 might be utilized as a potential biomarker and a putative therapeutic target, using either direct Gal‐1 inhibitors or small molecule PIM kinase inhibitors. Further studies directly testing these hypotheses are being developed.

## Conflict of interest

PR has received advisory board membership and lecture honoraria from Roche, BMS, and Pierre Fabre. PJ and IL have received research funding from Roche.

## Author contributions

PG designed the research, performed the research, analyzed the data, and wrote the manuscript. MW‐J designed the research, acquired funding, performed the research, analyzed the data, and wrote the manuscript. IL collected clinical information and analyzed the data. PR collected clinical information and analyzed the data. AP provided critical reagents. MS analyzed the data. PJ designed the research, analyzed the data, and wrote the manuscript.

## Supporting information


**Methods S1.** Phospho‐specific flow cytometry; Jurkat T cell activation assay.
**Table S1.** Primers used in gene expression analysis and plasmid generation.
**Table S2.** Antibodies used in WB and Flow cytometry.
**Table S3.** Melanoma patients enrolled in the study and their follow up. Corresponding Gal‐1 concentrations are indicated in Figure 6.
**Table S4.** (a‐d) Densitometric quantification of band intensities.
**Fig. S1.** PD‐L1 expression after 24 h incubation with cobimetinib or trametinib alone and in combination with IFN‐γ.
**Fig. S2.** ERK phosphorylation after 24 h incubation with vemurafenib in A375, MEL5 and MEL28 melanoma cell lines, analyzed by flow cytometry.
**Fig. S3.** Expression of CD25 and CD69 activation markers in Jurkat T cells after 24h coculture with A375 cells, pretreated with IFN‐γ alone or IFN‐γ in combination with vemurafenib for 24h.
**Fig. S4.** Relative Gal‐1 mRNA (A) and protein (B) expression in A375 and MEL28 melanoma cell lines after 24h incubation with vemurafenib.
