# Supplementary material for: BRAF inhibition curtails IFN‐gamma‐inducible PD‐L1 expression and upregulates the immunoregulatory protein galectin‐1 in melanoma cells
Source: Mol Oncol. 2020 May 19;14(8):1817–32. doi: 10.1002/1878-0261.12695 (PMC7400781; doi:10.1002/1878-0261.12695)

**Supplemental information**

**BRAF inhibition curtails IFN-gamma inducible PD-L1 expression and upregulates immunoregulatory protein, galectin-1 in melanoma cells.**

Patryk Gorniak^1^, Maja Wasylecka-Juszczynska^1^, Iwona Lugowska^2,3,4^, Piotr Rutkowski^2^, Anna Polak^1^, Maciej Szydlowski^1^, Przemyslaw Juszczyński^1*^

^1^ Department of Experimental Hematology, Institute of Hematology and Transfusion Medicine, Warsaw, Poland;

^2^ Department of Soft Tissue/Bone Sarcoma and Melanoma, Maria Sklodowska-Curie National Research Institute of Oncology, Warsaw, Poland ;

^3^ Department of Biostatistics, Institute of Mother and Child, Warsaw, Poland;

^4^ Early Phase Clinical Trial Unit, Maria Sklodowska-Curie National Research Institute of Oncology, Warsaw, Poland.

**Supplemental Methods**

**Phospho-specific flow cytometry**

### Activity of ERK was assessed using intracellular phospho-specific flow cytometry according to manufacturer’s protocol (BD Phosflow Protocol II, BD Biosciences). Briefly, 1×10^6^ cells were resuspended in 1 mL of ice cold PBS, then fixed, permeabilized and stained with phycoerythrin conjugated phospho-ERK (Thr202/Tyr204) or isotype control antibody (IgG1ĸ) (BD Bioscience). Flow cytometry analysis was performed on FACS Canto flow cytometer (BD Biosciences) and FlowJo v.10 software.

### Jurkat T cell activation assay

A375 cells plated on 24-well plate were treated with IFN-γ (100 ng/uL) alone or in combination with vemurafenib (2.5 µM) for 24 hours. Jurkat T cells were stimulated for 24 hours, using human T-activator CD3/CD28 Dynabeads (Thermo Scientific) and 1x10^5^ cells were mixed with pretreated A375. After 24 hours, activation markers (CD25 and CD69) of Jurkat T-cells were assessed in cocultures by flow cytometry. Cells were stained with APC-CD7 antibody to discriminate T cells from A375 cells (T cells: CD7-positive; A375 cells: CD7-negative) and subsequently with PE-CD25 and PE-CD69 antibodies. CD25 and CD69 expression were evaluated in CD7-positive population using BD FACS Canto flow cytometer (BD Biosciences) and analyzed by FlowJo v.10 software.

**Supplemental Table 1**. Primers used in gene expression analysis and plasmid generation.

| For_PDL1 | 5'-CATACAACAAAATCAACCAAAGA-3' |
| --- | --- |
| Rev_PDL1 | 5'-TGGTCTTACCACTCAGGACT-3' |
| For_GAL1 | 5'-TCGCCAGCAACCTGAATCTC-3' |
| Rev_GAL1 | 5'-GCACGAAGCTCTTAGCGTCA-3' |
| For_GAPDH | 5’-AGCCTCCCGCTTCGCTCTCT-3’ |
| Rev_GAPDH | 5’-CGACCAAATCCGTTGACTCCGAC-3’ |
| For_PDL1_FLAG_expr | 5'-GGAGCCATCTTATTATGCCTTG -3' |
| Rev_PDL1_FLAG_expr | 5'-CTTGTCATCGTCGTCCTTGTAGT-3' |
| F1_PDL1_FLAG | 5'-ATGAGGATATTTGCTGTCTTTATATTC -3' |
| R1_PDL1_FLAG | 5'-CGTCTCCTCCAAATGTGTATCACTTT-3' |
| F2 _PDL1_FLAG | 5'-ACTGGCCGGCATGAGGATATTTGCTGTCTT-3' |
| R2_PDL1_FLAG | 5'-ACTGGAATTCTTACTTGTCATCGTCGTCCTTGTAGTCCGTCTCCTCCAAATGTGTATCACTTT-3' |

**Supplemental Table 2**. Antibodies used in WB and Flow cytometry

| Antigen | Source |  |
| --- | --- | --- |
| Phospho-4E-BP1 (S65) | Cell Signaling; #9456 | WB |
| 4E-BP1 | Cell Signaling; #9452 | WB |
| Phospho-Erk (Thr202/Tyr204) | Cell Signaling; #4370 | WB |
| ERK 1/2 | Milipore; #05–1152 | WB |
| Phospho-p90RSK(T359/S363) | Cell Signaling; #9344 | WB |
| RSK1/2/3 | Cell Signaling; #9355 | WB |
| GAPDH | Milipore; #MAB 374 | WB |
| phospho-S6 (S235/236) | Cell Signaling; #4858 | WB |
| S6 | Cell Signaling; #2217 | WB |
| Stat1 | Cell Signaling; #9172 | WB |
| Phospho-Stat1 (Y701) | Cell Signaling; #9167 | WB |
| Phospho-Stat1 (YS727) | Cell Signaling; #8826 | WB |
| Gal-1 | Cell Signaling; #5418 | WB |
| PD-L1 | Cell Signaling; #13684 | WB |
| FLAG | Sigma-Aldrich; #F1804 | WB |
| PE-PD-L1 | Biolegend; #329706 | FACS |
| APC-CD7 | BD; #561604 | FACS |
| PE-CD25 | BD; #557138 | FACS |
| PE-CD69 | BD; #560968 | FACS |
| PE-IgG2b,k | Biolegend; #400313 | FACS |
| PE-Phospho-Erk (Thr202/Tyr204) | BD Bioscience; #612566 | FACS |
| PE-IgG1ĸ | BD Bioscience;# 559320 | FACS |

### Supplemental Table 3. Melanoma patients enrolled in the study and their follow up. Corresponding Gal-1 concentrations are indicated in Figure 6.

| Patients | Age | Gender | Start date | Type of treatment | Date (1) of assessment | RR  (1) | Date (2) of assessment | RR  (2) | |
| --- | --- | --- | --- | --- | --- | --- | --- | --- | --- |
| 701769 | 42 | male | 13/03/2019 | DT | 12/07/2019 | SD | 03/10/2019 | PR | |
| 934668 | 54 | male | 08/06/2018 | DT | 03/08/2019 | PD | n/a | n/a | |
| 1011339 | 38 | male | 01/03/2019 | DT | 14/06/2019 | CR | n/a | CR | |
| 1347511 | 62 | female | 11/01/2019 | DT | 07/06/2019 | PR | 13/08/2019 | PR | |
| 1357177 | 80 | male | 24/05/2019 | DT | 12/07/2019 | PR | 20/09/2019 | PR | |
| 1288520 | 63 | female | 16/02/2018 | DT | 14/05/2018 | PR | 20/08/2018 | PR | |
| 1256734 | 64 | male | 13/04/2018 | DT | 15/06/2018 | SD | 10/08/2018 | SD* | |
| 1274342 | 43 | male | 13/03/2018 | DT | 28/06/2018 | SD | 26/10/2018 | PR | |
| 1167615 | 61 | male | 21/12/2018 | DT | 25/01/2019 | SD | 22/03/2019 | SD | |
| * at that time patient had one metastatic brain lesion (size: 7 mm), which was treated with stereotactic body radiation therapy (SBRT); other site of metastases were stable acc.to RECIST 1.1; RR, radiological response; PD, progressive disease; SD, stable disease; PR, progression; CR, complete remission; DT, dabrafenib + trametinib | | | | | | | | |  |

### Supplemental Tables 4a-d. Densitometric quantification of band intensities in western blots included in Figure 2B, 2C, 3A, 3B.

### Supplemental Table 4a (to Figure 2B)

|  | A375 | | | MEL28 | | |
| --- | --- | --- | --- | --- | --- | --- |
|  | **DMSO** | **IFN 100ng/mL** | **Vem 2.5uM**  **IFN 100ng/mL** | **DMSO** | **IFN 100ng/mL** | **Vem 2.5uM**  **IFN 100ng/mL** |
| p-STAT1(S727) | **-** | **1.00** | **0.42** | **-** | **1.00** | **0.30** |
| p-STAT1(Y701) | **-** | **1.00** | **0.47** | **-** | **1.00** | **0.63** |
| STAT1 | **-** | **1.00** | **1.85** | **-** | **1.00** | **0.72** |
| p-ERK (T202/Y204) | **-** | **1.00** | **0.06** | **-** | **1.00** | **0.05** |
| ERK | **-** | **1.00** | **0.52** | **-** | **1.00** | **0.92** |

Acquired band intensities of p-ERK (T202/Y204), p-STAT1(S727) and p-STAT1(Y701) were normalized to ERK and STAT1 respectively (set to an arbitrary value 1.00) and presented as fold change. ERK and STAT1 band intensities were normalized to a housekeeping control (GAPDH) and presented as fold change. Baseline conditions were set to an arbitrary value 1.00.

### Supplemental Table 4b (to Figure 2C)

| A375_MEK_Q56P | | | | | | | A375_MEK_wt | | |
| --- | --- | --- | --- | --- | --- | --- | --- | --- | --- |
|  | **DMSO** | **Vem 2.5uM** | **IFN 100ng/mL** | **Vem 2.5uM**  **IFN 100ng/mL** | **DMSO** | **Vem 2.5uM** | | **IFN 100ng/mL** | **Vem 2.5uM**  **IFN 100ng/mL** |
| p-STAT1 (S727) | **-** | **-** | **1.00** | **1.61** | **-** | **-** | | **1.00** | **0.58** |
| p-STAT1 (Y701) | **-** | **-** | **1.00** | **0.96** | **-** | **-** | | **1.00** | **0.62** |
| STAT1 | **-** | **-** | **1.00** | **1.30** | **-** | **-** | | **1.00** | **0.84** |
| p-ERK (T202/Y204) | **-** | **-** | **1.00** | **0.91** | **-** | **-** | | **1.00** | **0.39** |
| ERK | **-** | **-** | **1.00** | **1.23** | **-** | **-** | | **1.00** | **0.96** |

Acquired band intensities of p-ERK (T202/Y204), p-STAT1(S727) and p-STAT1(Y701) were normalized to ERK and STAT1 respectively (set to an arbitrary value 1.00) and presented as fold change. ERK and STAT1 band intensities were normalized to a housekeeping control (GAPDH) and presented as fold change. Baseline conditions were set to an arbitrary value 1.00.

### Supplemental Table 4c (to Figure 3A).

| A375 | | | | | MEL28 | | | | |
| --- | --- | --- | --- | --- | --- | --- | --- | --- | --- |
|  | **DMSO** | **Vem 0.5uM** | **Vem 2.5uM** | **Vem 10uM** | | **DMSO** | **Vem 0.5uM** | **Vem 2.5uM** | **Vem 10uM** |
| p-ERK (T202/Y204 | **1.00** | **0.00** | **0.00** | **0.06** | | **1.00** | **0.14** | **0.03** | **0.00** |
| ERK | **1.00** | **0.77** | **1.13** | **0.73** | | **1.00** | **1.09** | **1.21** | **1.00** |
| p-90RSK (T359/S363) | **1.00** | **0.00** | **0.00** | **0.00** | | **1.00** | **0.00** | **0.00** | **0.00** |
| 90RSK | **1.00** | **0,36** | **0,68** | **0,34** | | **1.00** | **1.11** | **1.02** | **0.65** |
| p-S6 (S235/236) | **1.00** | **0.00** | **0.00** | **0.00** | | **1.00** | **0.00** | **0.00** | **0.00** |
| S6 | **1.00** | **0.81** | **1.04** | **0.77** | | **1.00** | **0.90** | **0.91** | **0,79** |
| p-4E-BP1 (S65) | **1.00** | **0.06** | **0.22** | **0.09** | | **1.00** | **0.19** | **0.36** | **0.12** |
| 4E-BP1 | **1.00** | **0.96** | **0.89** | **0.83** | | **1.00** | **1.03** | **1.06** | **0.90** |

Acquired band intensities of p-ERK (T202/Y204), p-90RSK (T359/S363), p-S6 (S235/236) and p-4E-BP1 (S65) were normalized to ERK, 90RSK, S6 and 4E-BP1 respectively (set to an arbitrary value 1.00) and presented as fold change. ERK, 90RSK, S6 and 4E-BP1 band intensities were normalized to a housekeeping control (GAPDH) and presented as fold change. Baseline conditions were set to an arbitrary value 1.00.

### Supplemental Table 4d (Figure 3B)

| A375 | | | | | | | MEL28 | | |
| --- | --- | --- | --- | --- | --- | --- | --- | --- | --- |
|  | **DMSO** | **Vem 2.5uM** | **IFN 100ng/mL** | **Vem 2.5uM**  **IFN 100ng/mL** | **DMSO** | **Vem 2.5uM** | | **IFN 100ng/mL** | **Vem 2.5uM**  **IFN 100ng/mL** |
| p-ERK (T202/Y204) | **1.00** | **0.13** | **1.00** | **0.08** | **1.00** | **0.04** | | **1.00** | **0.02** |
| ERK | **1.00** | **1.08** | **1.00** | **0.89** | **1.00** | **1.03** | | **1.00** | **1.07** |
| p-90RSK (T359/S363) | **1.00** | **0.36** | **1.00** | **0.49** | **1.00** | **0.34** | | **1.00** | **0.27** |
| 90RSK | **1.00** | **0.86** | **1.00** | **0.80** | **1.00** | **1.01** | | **1.00** | **0.79** |
| p-S6 (S235/236) | **1.00** | **0.10** | **1.00** | **0.12** | **1.00** | **0.18** | | **1.00** | **0.00** |
| S6 | **1.00** | **1.04** | **1.00** | **1.01** | **1.00** | **1.59** | | **1.00** | **0.78** |
| p-4E-BP1 (S65) | **1.00** | **0.36** | **1.00** | **0.54** | **1.00** | **0.49** | | **1.00** | **0.70** |
| 4E-BP1 | **1.00** | **0.75** | **1.00** | **0.59** | **1.00** | **0.87** | | **1.00** | **0.67** |

Acquired band intensities of p-ERK (T202/Y204), p-90RSK (T359/S363), p-S6 (S235/236) and p-4E-BP1 (S65) were normalized to ERK, 90RSK, S6 and 4E-BP1 respectively (set to an arbitrary value 1.00) and presented as fold change. ERK, 90RSK, S6 and 4E-BP1 band intensities were normalized to a housekeeping control (GAPDH) and presented as fold change. Baseline conditions were set to an arbitrary value 1.00.

**Figure S1.** PD-L1 expression after 24h incubation with cobimetinib or trametinib alone and in combination with IFN-γ. Cells were pretreated with inhibitor for 2 h and then incubated with IFN-γ for 24h. Flow cytometry analysis.


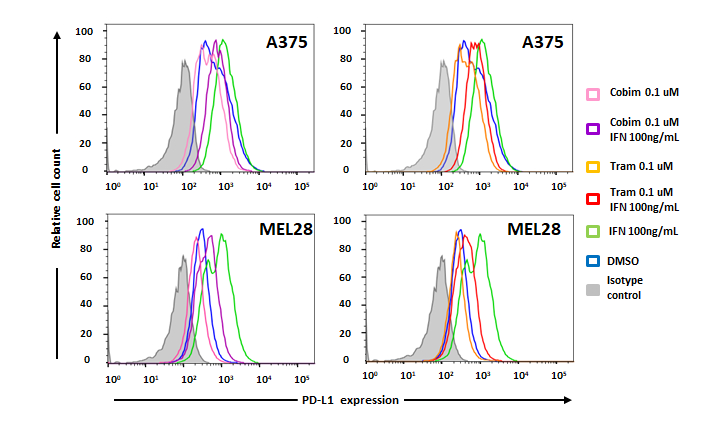


**Figure S2.** ERK phosphorylation after 24h incubation with vemurafenib in A375, MEL5 and MEL28 melanoma cell lines, analyzed by flow cytometry.


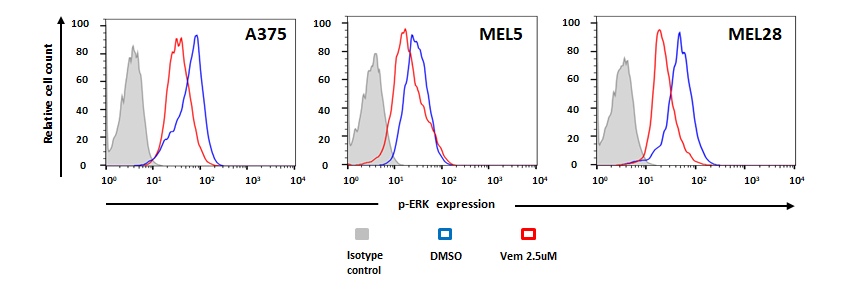


**Figure S3.** Expression of CD25 and CD69 activation markers in Jurkat T cells after 24h coculture with A375 cells, pretreated with IFN-γ alone (blue line) or IFN-γ in combination with vemurafenib (red line) for 24h.

**
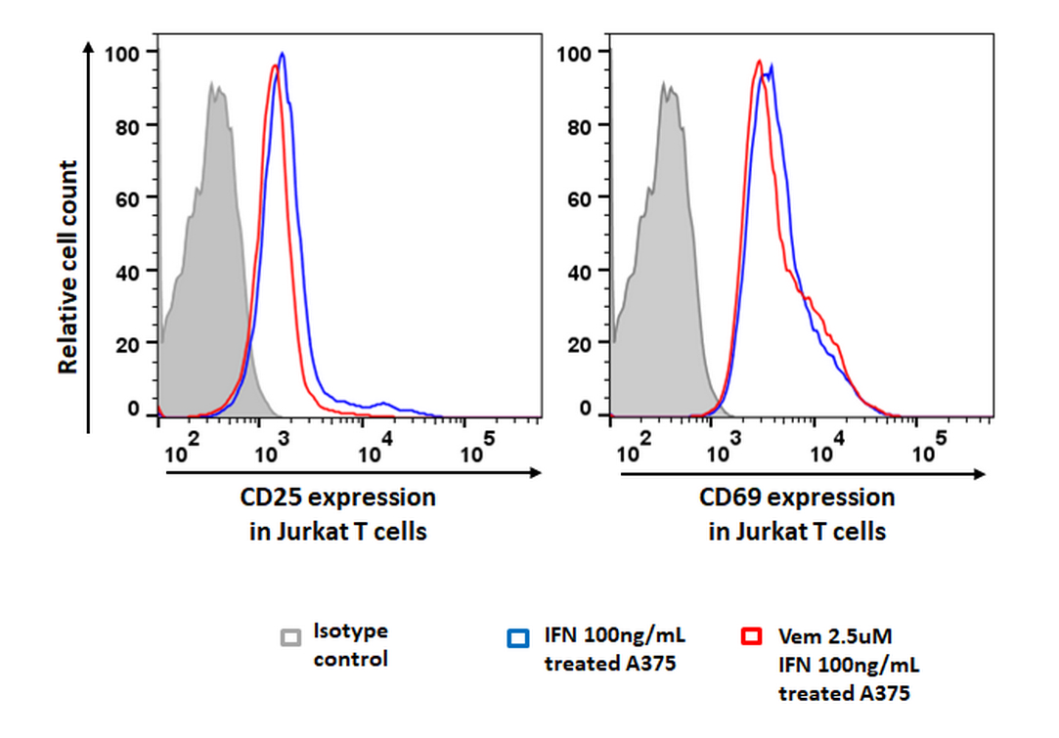
**

**Figure S4.** Relative Gal-1 mRNA (A) and protein (B) expression in A375 and MEL28 melanoma cell lines after 24h incubation with vemurafenib.


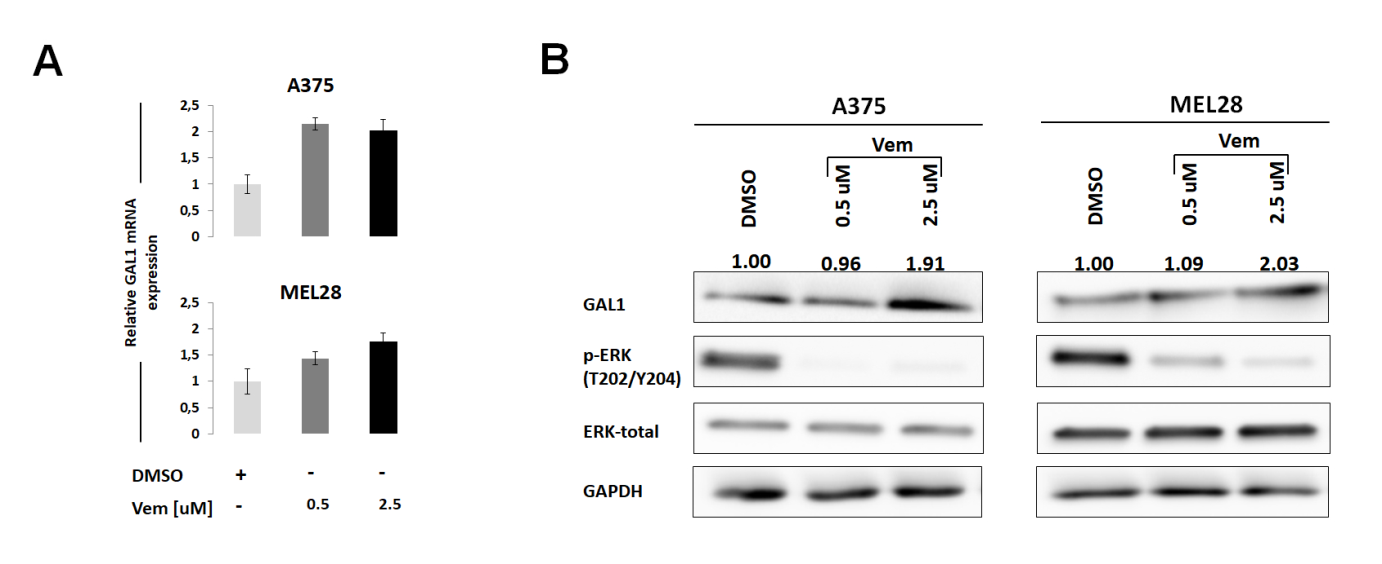

Supplement: Supplementary file 1 — Methods S1. Phospho‐specific flow cytometry; Jurkat T cell activation assay. Table S1. Primers used in gene expression analysis and plasmid generation. Table S2. Antibodies used in WB and Flow cytometry. Table S3. Melanoma patients enrolled in the study and their follow up. Corresponding Gal‐1 concentrations are indicated in Figure 6. Table S4. (a‐d) Densitometric quantification of band intensities. Fig. S1. PD‐L1 expression after 24 h incubation with cobimetinib or trametinib alone and in combination with IFN‐γ. Fig. S2. ERK phosphorylation after 24 h incubation with vemurafenib in A375, MEL5 and MEL28 melanoma cell lines, analyzed by flow cytometry. Fig. S3. Expression of CD25 and CD69 activation markers in Jurkat T cells after 24h coculture with A375 cells, pretreated with IFN‐γ alone or IFN‐γ in combination with vemurafenib for 24h. Fig. S4. Relative Gal‐1 mRNA (A) and protein (B) expression in A375 and MEL28 melanoma cell lines after 24h incubation with vemurafenib. [file MOL2-14-1817-s001.docx]
